# Screening for Depression in Low-Income Elderly Patients at the Primary Care Level: Use of the Patient Health Questionnaire-2

**DOI:** 10.1371/journal.pone.0113778

**Published:** 2014-12-04

**Authors:** Valéria Teresa Saraiva Lino, Margareth Crisóstomo Portela, Luiz Antônio Bastos Camacho, Soraya Atie, Maria José Barbosa Lima, Nádia Cristina Pinheiro Rodrigues, Mônica Bastos de Lima Barros, Mônica Kramer de Noronha Andrade

**Affiliations:** National Public Health School/Oswaldo Cruz Foundation, Rio de Janeiro, Brazil; University of South Florida, United States of America

## Abstract

**Introduction:**

Depression is one of the most common mental disorders and a leading cause of disability worldwide. It constitutes a serious public health problem, particularly among elderly individuals. Most depressed elderly patients are treated by primary care (PC) physicians. The “Patient Health Questionnaire” (PHQ-2) is an instrument used for the detection of depression in PC settings.

**Objective:**

Evaluate the performance of the PHQ-2 in a low-income and uneducated elderly PC population.

**Methods:**

A non-probabilistic population sample of 142 individuals was selected from the healthcare unit's users ≧60 years. Criterion validity was assessed by estimating the sensitivity, specificity, positive predictive value (PPV), and negative predictive value (NPV) of the PHQ-2 in comparison with the structured interview using the DSM-IV. The estimates of sensitivity and specificity were obtained from varying cut-offs of the PHQ-2 score. A Receiver Operator Characteristic (ROC) curve was constructed and the area under the curve (AUC) was calculated.

**Results:**

The group was predominantly female (73.9%), with low education level (mean 3 years of schooling). The mean age was 72.5 years old. The prevalence of depression was 26.1%. The best values of sensitivity (0.74), specificity (0.77), PPV (0.50) e NPV (0.90) were obtained with score equal to 1. The AUC was 0.77, indicating a modest performance of the test accuracy.

**Conclusion:**

The simplicity of the PHQ-2 is an advantage for its use in PC. The high NPV indicated that 90% of those who tested negative would not need additional tests. However, the low PPV indicated that the PHQ-2 is not sufficient to screen for depression. The application of the instrument could be the first step of the screening, that would include a second step to all those with positive tests formerly.

## Introduction

Depression is one of the most common mental disorders. This leading cause of disability worldwide constitutes a serious public health problem [1], particularly among elderly individuals. The prevalence of depressive symptoms in people aged 60 and over living in communities in Brazil varies from 22% to 35% [2]. Depression is more common in women. Physicians from a wide variety of medical specialties treat patients affected by depression, but most depressed elderly patients are treated by primary care (PC) physicians. Follow-up by health care professionals in the PC setting provides the opportunity to track depression over time, which is an advantage because of the frequent recurrence and chronicity associated with the disorder [3]. Nevertheless, only 40–50% of cases of depression are detected in PC, and only 20% of these cases receive appropriate treatment [4].

A consensus about whether screening for depression should occur in PC practices has not been reached. The United States Preventive Services Task Force recommends that patients should be screened when there is an integrated mental health service system that enables the management of the disease [5]. However, the United Kingdom's National Institute for Health and Care Excellence noted a lack of evidence that depression screening would benefit patients [6]. The American Heart Association recommends that patients with coronary heart disease should be screened for depression [7], while Thombs *et al.* has questioned it claiming that there are available few sensitive and specific screening instruments. They also found that treatment with antidepressants or psychotherapy leads to only a slight reduction in depressive symptoms [8].

The potential negative outcomes that can result from routine screening for depression include the need to treat misdiagnosed patients, the treatment of mild symptoms that would resolve without intervention and, perhaps most importantly, the division of scarce resources that could be used to ensure better care for patients already identified with depression [9]. Despite these risks, we must consider that the current status of the low quality of the management of depression results in serious public health problems. Souminent et al. investigated 1,198 attempted suicides in Finland and found that the vast majority of the older people had contact with health professionals in the 12 months prior to the event [10]. However, only 4% had received a diagnosis of mood disorder before the suicide attempt occurred. This type of negative outcome indicates that training in the identification and treatment of depression are important PC issues. Most of the individuals in the elderly population visit their PC physician each year, so these professionals are in an excellent position to improve the diagnosis and management of depressive disorders [11].

The concept of mental health literacy provides another justification for screening for depression in PC, particularly the low-income elderly. Mental health literacy encompasses the ability to recognize mental illness, the knowledge and beliefs about causes, self-help, and professional help. Lack of mental health literacy contributes to low rates of recognition of problems, delays in seeking help, and may be more prevalent among individuals in lower socioeconomic groups [1].

The “Patient Health Questionnaire” (PHQ-9) is a survey instrument used for the detection of depression in PC settings [12]. It consists of nine questions and allows for the quantification of discomfort levels and for the classification of disease severity. A reduced version with two questions (PHQ-2) that ask about the frequency of depressed mood and anhedonia (loss of sense of pleasure in performing acts that were previously pleasurable) in the previous 2 weeks has a reported sensitivity of 83% and specificity of 92% [13]. The PHQ-2 has been evaluated for screening for depression in the elderly, and the authors concluded that its use is an appropriate first step for screening for depression in elderly individuals [14], [15]. The validity of the PHQ-2 in Brazil has been confirmed for different settings. In the context of PC, Osorio et al. has identified sensitivity and specificity of 97% and 88%, respectively, in a sample of women referred to the gynecology and general practice services [16]. In patients admitted to the clinical wards of a general university hospital, the instrument demonstrated good accuracy, with an area under the ROC curve of 0.89 (p<0.0001) [17]. And in a movement disorders outpatient clinic, its sensitivity and specificity were 75% and 89%, respectively [18].

The simplicity and the good performance of the PHQ-2 in terms of the psychometric properties among Brazilian people, enable the performance assessment of the instrument in elderly Brazilian users of primary care, where professionals are pressured by high demand for care. This work aimed to evaluate the performance of the PHQ-2 in an elderly PC population with low income and education status.

## Methods

### Study Setting and Sample

This research was part of an exploratory study that used a comprehensive geriatric assessment (CGA) of various patient functions. It aimed to develop a screening strategy for use in elderly patients visiting PC. The study was performed in a geriatric care setting at the PC unit of the National Public Health School, Oswaldo Cruz Foundation (Brazilian Ministry of Health, Rio de Janeiro, Brazil). The unit provides services to >31,000 residents of the Manguinhos District in the city of Rio de Janeiro. Most of the residents of this area live in houses with a single room and earn a monthly family income of approximately $270 USD per month, which is equivalent to 1.17 times the 2010 minimum wage [19]. More than 50% of the residents have not attended school beyond the elementary level. Initiatives aimed at decreasing violence have been impeded by the activities of local drug traffickers [20].

A non-probabilistic population sample was selected from the healthcare unit's users ≧60 years of age who received care from family physicians and the healthcare team. Individuals with advanced cognitive decline, blindness, deafness, or impaired locomotion were excluded from the original research. The sample size used in the original study was calculated using the prevalence of depression as a reference. Depression, estimated at 20% in the elderly, was one of the conditions targeted by the CGA because the largest sample size would be generated [14]. A sample size of 180 individuals (kappa coefficient of 0.6 and a 95% confidence interval of 0.1) was estimated using WinPepi statistical software [21]. Because the objective of our study was to validate the use of the PHQ-2, we excluded the volunteers who experienced cognitive decline after the evaluation that used the CGA. The final sample size consisted of 142 individuals.

### Procedures

Data collection occurred from June to December 2010. For the diagnosis of major depression, a physician trained in the treatment of depression applied the Structured Clinical Interview for DSM-IV Axis I Disorders (SCID-I), Clinician Version [22]. Its test-retest reliability has been verified in Brazil, with good results for diagnosis of major depression (kappa = 0.87) [23]. The screening using the PHQ-2 was performed by a social worker or a psychomotor specialist, both professionals of the PC clinic, 7–15 days after the CGA. The PHQ-2 is presented in this form: “Over the past 2 weeks, how often have you been bothered by any of the following problems: (1) little interest or pleasure in doing things? (2) feeling down, depressed, or hopeless?” The score for each question ranges from zero (no days) to three (almost every day) [13]. The overall score for the PHQ-2 ranges from zero to six, with increasing severity of depression as the value increases. The results of a previous study indicated that a score ≧3 indicates 83% sensitivity and 92% specificity for major depression [13].

The version translated into Brazilian Portuguese was used in this study. [24] A pre-test revealed that the respondents had a better understanding of the two questions when each one was broken in two parts, first capturing the presence of the event, and after the frequency in which it occurred. So, each interviewee answered the question about whether sadness or anhedonia had occurred during the previous 2 weeks, and then answered the questions about frequencies of occurrence (“Over the past 2 weeks, have you been bothered by any of the following problems?…”; “how often?”) This change resulted in the use of four sentences during the assessment, but it was recorded only the highest score achieved using one of two sentences of each item. We thus maintained the definition of the original score.

### Data analysis

Criterion validity was assessed by estimating the sensitivity, specificity, positive predictive value (PPV), and negative predictive value (NPV) of the PHQ-2 in comparison with the structured interview using the DSM-IV.

A simple logistic model was used for the prediction of depression, which was defined using the SCID-I. The PHQ-2 score was the predictor. The estimates of sensitivity and specificity were obtained from varying cut-offs of the PHQ-2 score. A Receiver Operator Characteristic (ROC) curve was constructed and the area under the curve was calculated. The optimum pair of sensitivity and specificity values was indicated by the point at which the ROC value was closest to the upper left corner of the curve. In addition to the visual analysis, we used the Youden index (sensitivity+specificity-1) to determine the best cutoff value for the model. This index was used to provide an assessment of sensitivity and specificity that assumed that both were equally important. SPSS version 18 statistical software was used for the analyses.

### Ethics Statement

The study was approved by the Research Ethics Committee of the Sergio Arouca National School of Public Health, Oswaldo Cruz Foundation (FIOCRUZ; report number 126/10). All participants signed the informed voluntary consent from, and protection of the confidentiality of collected data was maintained.

## Results

One hundred forty-two individuals without cognitive decline participated in the validation of the PHQ-2. The group was predominantly female and consisted of individuals with low education levels (Table 1) Only seven people had studied beyond the elementary school. Respondents were more likely to be single or widows/widowers than they were to be married. Depression was very common among the elderly respondents.

**Table 1:** Characteristics of the study participants (N = 142).

Variable	Mean	SD
**Age (years)**	72.5	[7.0]
**Level of education (years)**	3	[3.0]

	n	%
**Sex**		
**Female**	105	73.9
**Male**	37	26.1
**Marital status**		
**Married**	69	48.6
**Single/widow(er)**	73	51.3
**Depression (SCID-I)**	37	26.1

**Figure 1:**
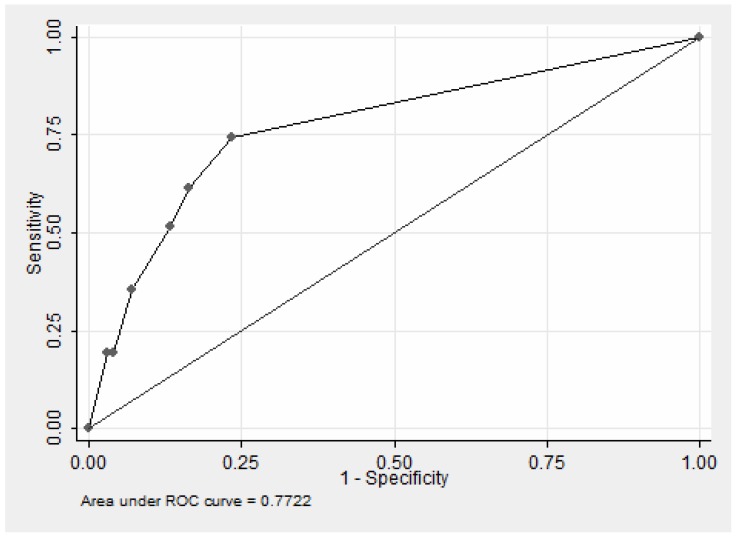
ROC curve of predictive logistic regression model for depression according to the scores of the PHQ – 2.

The results of the analysis of the psychometric properties of the PHQ-2 indicated that the values for sensitivity declined as the score value increased. (Table 2; Figure 1). For these values, the PPV indicated that depression in 50% of those with a positive test was not confirmed. However, the corresponding result of NPV = 0.90 indicated that 10% of those with negative test results had a false negative diagnosis of depression.

**Table 2:** Psychometric properties of the PHQ-2, by score cut-off.

Score	Sensitivity	Specificity	PPV	NPV
1	0.74	0,77	0.50	0.90
2	0.61	0,84	0.54	0.87
3	0.52	0,87	0.55	0.85
4	0,35	0.93	0.61	0.92
5	0.19	0.96	0.60	0.69
6	0.19	0.97	0.67	0.79

The area under the curve (Figure 1) was 0.77, indicating a modest performance of the test accuracy.

## Discussion

The high prevalence of depression in this study population is consistent with the results of previous Brazilian studies. In a systematic review, Barcellos-Ferreira et al. found that clinically significant depressive symptoms (CSDSs) occur in 26% of community-dwelling elderly individuals [25]. However, the prevalence of CSDSs has been reported to be as high as 45% among subjects in outpatient care settings [26]. The values for the prevalence of depression reported in these studies are even higher than the values reported in international studies. Luppa et al. reported that the results of their systematic review and meta-analysis of depression prevalence indicated that depressive disorders occur in 17% of elderly individuals [27]. Steffens et al. found an overall prevalence of depression of 11.2% in a representative USA population sample [28].

The large variation in depression prevalence worldwide might be due to differences in measurement methods and varying contributions of social, cultural, and economic risk factors. Socioeconomic position, stressful life events, violence, education levels, and income inequality are determinants of depression [1]. Gender inequity is also a determinant, and increases the risk of depression among women. All of these factors may have contributed to the results of our study, because the study population consisted exclusively of elderly, predominantly female, individuals who resided in a region of low socioeconomic status. Local violence contributed to increased levels of stress on the residents. Increased values for the prevalence of depressive symptoms in groups of mostly elderly, female individuals with low levels of education and income have also been reported by other Brazilian investigators [26], [29].

### Criterion validity of the PHQ-2

The values for sensitivity and specificity obtained in assessing the criterion validity of the PHQ-2 were not encouraging. However, this screening process had a high NPV (90%) when used for a diagnosis of depression. These results are consistent with the results reported by Phelan et al. [30]. In their study of 71 seniors in PC, the values for sensitivity and specificity were 0.75 and 0.67, respectively. The sample size used in our study might have contributed to a reduction in test accuracy. Using a sample of 580 patients from PC services, Kroenke et al. obtained sensitivity and specificity values of 0.83 and 0.90, respectively [11]. Arrol et al. interviewed 2,642 individuals using PC services and measured a sensitivity of 0.86 and a specificity of 0.78 [11], [13].

The low educational level of the study population was an additional factor that may have affected the results for PHQ-2 sensitivity. According to the interviewers, the elderly respondents indicated that it was difficult to understand the response options for the questionnaire. The effect of educational level on test sensitivity was examined by Cutler et al. in a study of maternal depression in a low-educational level population. The sensitivity and specificity for the PHQ-2 was 0.43 and 0.97, respectively, but the sensitivity was higher for women who were educated beyond high school, compared with those who were not [31].

The previous examination of the psychometric characteristics of the PHQ - 2 in Brazil resulted in satisfactory levels when it was tested in hospital settings and outpatient specialty clinics, with samples involving adults and seniors, but the best results were obtained in PC with women under age 50, when the sensitivity and specificity were equal to 0.97 and 0.88, respectively. In this study, most of women In this study, in which almost 70% of respondents had studied less than five years, low education did not reduced the performance of the PHQ – 2. [16].

Considering the modest performance illustrated by the ROC curve, use of the PHQ-2 resulted in considerable potential for misclassification. The PHQ-2 score cutoffs identified in our study differed from cutoffs reported by others in studies of the elderly [30] and of individuals with low educational levels [31], including in PC settings [11]. The items in the PHQ-2 represent core features of depression. The high NPV obtained by the PHQ-2 among elderly with low income and low education in the context studied indicated that 90% of those who tested negative would be correctly considered free of depression and not in need of confirmatory tests. However, the low PPV also indicated that the PHQ-2 is not sufficient to screen for depression. In this sense, the authors suggest the application of PHQ-2 as the first step of the screening, that would include a second step to all those with positive tests formerly. Considering that each question was broken into two sentences, making them clearer, and that the sentences are very direct, we believe that there would not be ways of improving significantly the performance of PHQ-2. Our appreciation is that its unsatisfactory performance is not due to the ways they are phrased or formatted, but due to cognitive features and health illiteracy of the population focused.

There were some limitations of this study. The sample population was selected from a medical setting, which might have resulted in an overestimate of the prevalence of depression. The clinical spectrum of the disorder in patients approached in the PC settings may make the differentiation of depressed and non-depressed subjects easier than in the community.

## Conclusion

PC may often be the first contact that individuals have with health services. In this setting, individuals are more likely to be seeking care during the early stage of a disorder. Distinct clinical signs and symptoms may be absent, and selective screening may be justified in high risk groups as the elderly. The simplicity of the PHQ-2 makes it useful as a first step in the screening of depression in typical PC units in which elderly patients with socioeconomic profiles that are similar to the profiles of the study population will be found. This process would include a second step to all those with positive tests formerly.

## References

[1] Patel V, Lund C, Hatherill S, Plagerson S, Corrigall J et al**.** (2010) Mental disorders: equity and social determinants. In: Blas E, Kurup AS, editors. Equity, social determinants and public health programmes. Geneva: World Health Organization. pp. 115–134.

[2] Blay SL, Andreoli SB, Fillenbaum GG, Gastal FL (2007) Depression morbidity in later life: prevalence and correlates in a developing country. Am J Geriatr Psychiatry 15:790–799. 17698602 10.1097/JGP.0b013e3180654179

[3] Park M, Unutzer J (2011) Geriatric depression in primary care. Psychiatr Clin North Am 34:469–487. 21536169 10.1016/j.psc.2011.02.009 PMC3184156

[4] Mitchell AJ, Rao S, Vaze A (2010) Do primary care physicians have particular difficulty identifying late-life depression? A meta-analysis stratified by age. Psychother Psychosom 79:285–294. 20616623 10.1159/000318295

[5] U.S. Preventive Services Task Force, Agency for Healthcare Research and Quality (2009) Screening for Depression in Adults: U.S. Preventive Services Task Force Recommendation Statement. Ann Intern Med 151(11):784–792. 19949144 10.7326/0003-4819-151-11-200912010-00006

[6] National Collaborating Center for Mental Health (2010) The NICE guideline on the management and treatment of depression in adults (updated edition). London (UK): National Institute for Health and Clinical Excellence. 706 p.

[7] Lichtman JH, Bigger JT Jr, Blumenthal JA, Frasure-Smith N, Kaufmann PG, et al (2008) Depression and coronary heart disease: recommendations for screening, referral, and treatment: a science advisory from the American Heart Association Prevention Committee of the Council on Cardiovascular Nursing, Council on Clinical Cardiology, Council on Epidemiology and Prevention, and Interdisciplinary Council on Quality of Care and Outcomes Research: endorsed by the American Psychiatric Association. Circulation 118:1768–1775. 18824640 10.1161/CIRCULATIONAHA.108.190769

[8] Thombs BD, Roseman M, Coyne JC, de Jonge P, Delisle VC, et al (2013) Does evidence support the American Heart Association's recommendation to screen patients for depression in cardiovascular care? An updated systematic review. PLoS One 8:e52654. 23308116 10.1371/journal.pone.0052654 PMC3538724

[9] Thombs BD, Coyne JC, Cuijpers P, de Jonge P, Gilbody S, et al (2012) Rethinking recommendations for screening for depression in primary care. CMAJ 184:413–418. 21930744 10.1503/cmaj.111035 PMC3291670

[10] Suominen K, Isometsa E, Lonnqvist J (2004) Elderly suicide attempters with depression are often diagnosed only after the attempt. Int J Geriatr Psychiatry 19:35–40. 14716697 10.1002/gps.1031

[11] Arroll B, Goodyear-Smith F, Crengle S, Gunn J, Kerse N, et al (2010) Validation of PHQ-2 and PHQ-9 to screen for major depression in the primary care population. Ann Fam Med 8:348–353. 20644190 10.1370/afm.1139 PMC2906530

[12] Spitzer RL, Kroenke K, Williams JB (1999) Validation and utility of a self-report version of PRIME-MD: the PHQ primary care study. Primary Care Evaluation of Mental Disorders. Patient Health Questionnaire. JAMA 282:1737–1744. 10568646 10.1001/jama.282.18.1737

[13] Kroenke K, Spitzer RL, Williams JB (2003) The Patient Health Questionnaire-2: validity of a two-item depression screener. Med Care 41:1284–1292. 14583691 10.1097/01.MLR.0000093487.78664.3C

[14] Li C, Friedman B, Conwell Y, Fiscella K (2007) Validity of the Patient Health Questionnaire 2 (PHQ-2) in identifying major depression in older people. J Am Geriatr Soc 55:596–602. 17397440 10.1111/j.1532-5415.2007.01103.x

[15] Richardson TM, He H, Podgorski C, Tu X, Conwell Y (2010) Screening depression aging services clients. Am J Geriatr Psychiatry 18:1116–1123. 20808102 10.1097/JGP.0b013e3181dd1c26 PMC2992075

[16] Osorio FL, Vilela MA, Crippa JA, Loureiro SR (2009) Study of the discriminative validity of the PHQ-9 and PHQ-2 in a sample of Brazilian women in the context of primary health care. Perspect Psychiatr Care 45:216–227. 19566694 10.1111/j.1744-6163.2009.00224.x

[17] Osorio FL, Carvalho AC, Fracalossi TA, Crippa JA, Loureiro ES (2012) Are two items sufficient to screen for depression within the hospital context? Int J Psychiatry Med 44:141–148. 23413661 10.2190/PM.44.2.e

[18] Chagas MH, Crippa JA, Loureiro SR, Hallak JE, Meneses-Gaya C, et al (2011) Validity of the PHQ-2 for the screening of major depression in Parkinson's disease: two questions and one important answer. Aging Ment Health 15:838–843. 21562987 10.1080/13607863.2011.569482

[19] Carvalho MAP, Pivetta F (2012) The integrated territory of health care in Manguinhos: we are all apprentices. Org. Rio de Janeiro: Escola Nacional de Saude Publica Sergio Arouca/Fiocruz. 183p.

[20] de Souza ER, Ribeiro AP, Atie S, de Souza AC, Marques CC (2008) The net for protection to the elderly of Rio de Janeiro: a right to be conquered. Cien Saude Colet 13:1153–1163. 18813614 10.1590/s1413-81232008000400011

[21] Abramson J (2009) WINPEPI (PEPI-for-Windows): computer programs for epidemiologists. 10.1186/1742-5573-1-6 PMC544871 15606913

[22] First MB, Spitzer RL, Gibbon M, Williams JBW (1996) Structured Clinical Interview for DSM-IV Axis I Disorders, Clinician Version (SCID-CV). Washington, D.C: American Psychiatric Press, Inc.

[23] Del-Ben CM, Vilela J, Crippa J, Hallak J, Labate C, et al (2001) Confiabilidade da Entrevista Clínica Estruturada para o DSM-IV - Versão Clínica traduzida para o portugues. Rev Bras Psiquiatr 23:156–159.

[24] Spitzer R, Williams J, Kroenke K (1999) The Patient Health Questionnaire-2 (PHQ-2)http://www.phqscreeners.com/pdfs/01_PHQ/PHQ_Portuguese%20for%20Brazil.pdf. Accessed 17 March 2010.

[25] Barcelos-Ferreira R, Izbicki R, Steffens DC, Bottino CM (2010) Depressive morbidity and gender in community-dwelling Brazilian elderly: systematic review and meta-analysis. Int Psychogeriatr 22:712–726. 20478096 10.1017/S1041610210000463

[26] Castro-Costa E, Lima-Costa M, Carvalhais S, Firmo J, Uchoa E (2008) Factors associated with depressive symptoms measured by the 12-item General Health Questionnaire in Community-Dwelling Older Adults (The Bambuí Health Aging Study). Rev Bras Psiquiatr 30:5. 10.1590/s1516-44462008005000007 18470408

[27] Luppa M, Sikorski C, Luck T, Ehreke L, Konnopka A, et al (2012) Age- and gender-specific prevalence of depression in latest-life–systematic review and meta-analysis. J Affect Disord 136:212–221. 21194754 10.1016/j.jad.2010.11.033

[28] Steffens DC, Fisher GG, Langa KM, Potter GG, Plassman BL (2009) Prevalence of depression among older Americans: the Aging, Demographics and Memory Study. Int Psychogeriatr 21:879–888. 19519984 10.1017/S1041610209990044 PMC2747379

[29] Almeida-Filho N, Lessa I, Magalhaes L, Araujo MJ, Aquino E, et al (2004) Social inequality and depressive disorders in Bahia, Brazil: interactions of gender, ethnicity, and social class. Soc Sci Med 59:1339–1353. 15246165 10.1016/j.socscimed.2003.11.037

[30] Phelan E, Williams B, Meeker K, Bonn K, Frederick J, et al (2010) A study of the diagnostic accuracy of the PHQ-9 in primary care elderly. BMC Fam Pract 11:63. 20807445 10.1186/1471-2296-11-63 PMC2940814

[31] Cutler CB, Legano LA, Dreyer BP, Fierman AH, Berkule SB, et al (2007) Screening for maternal depression in a low education population using a two item questionnaire. Arch Womens Ment Health 10:277–283. 17710366 10.1007/s00737-007-0202-z

